# Supplementary material for: Causal interplay between lactose intolerance and gut microbiota: a combined bidirectional Mendelian randomization and in vivo validation study
Source: Front Nutr. 2026 Jun 1;13:1803337. doi: 10.3389/fnut.2026.1803337 (PMC13265576; doi:10.3389/fnut.2026.1803337)
Supplement: Supplementary file 1 [file Data_Sheet_1.zip › supplementary materials/Forward/leave one out cross validation/ebi-a-GCST90027634.finngen_R12_E4_LACTONAS.pdf]

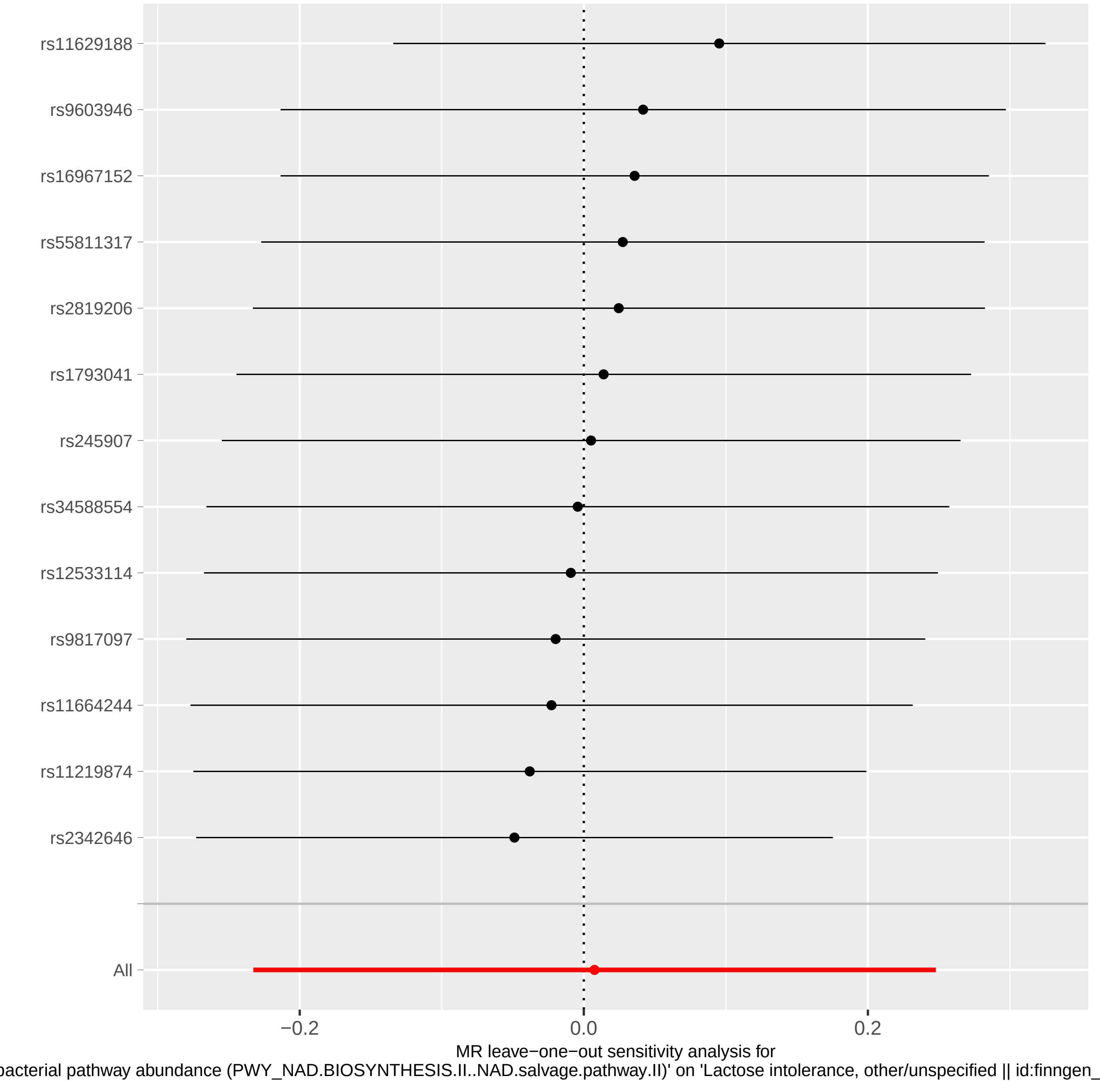
rs11629188
rs9603946
rs16967152
rs55811317
rs2819206
rs1793041
rs245907
rs34588554
rs12533114
rs9817097
rs11664244
rs11219874
rs2342646
All
−0.2
0.0
0.2
MR leave−one−out sensitivity analysis for
bacterial pathway abundance (PWY_NAD.BIOSYNTHESIS.II..NAD.salvage.pathway.II)' on 'Lactose intolerance, other/unspecified || id:finngen_
